# Evaluation of total LDH and its isoenzymes as markers in preeclampsia

**DOI:** 10.2478/jomb-2019-0045

**Published:** 2020-09-02

**Authors:** Fazal Rimsha Saleem, Swetha Chandru, Monalisa Biswas

**Affiliations:** 1 MVJ Medical College and Research Hospital, Department of Biochemistry, Hoskote, Bangalore, Karnataka, India; 2 Manipal Academy of Higher Education (MAHE), Kasturba Medical College, Department of Biochemistry, Manipal, Karnataka, India

**Keywords:** preeclampsia, eclampsia, lactate dehydrogenase, preeklampsija, eklampsija, laktat dehidrogenaza

## Abstract

**Background:**

Preeclampsia, a rapidly progressing pregnancy-specific multi-systemic syndrome is globally the leading cause of maternal and neonatal morbidity and mortality. This study aims to evaluate the serum total Lactate dehydrogenase levels in women with preeclampsia when compared to normotensive pregnant women and assess the electrophoretic pattern of the LDH isoenzymes in normal pregnancy, preeclampsia and eclampsia.

**Methods:**

The study, carried out in the Department of Biochemistry of MVJ Medical College, included 30 patients of preeclampsia and 30 normotensive gestational age-matched pregnant women admitted to the Department of OBG. Serum total LDH was analysed by DGKC method. Serum and cord blood samples for isoenzyme distribution analysis were collected from a normal pregnant woman undergoing delivery, a woman with mild eclampsia, two women with eclampsia, and analysed by slab gel electrophoresis followed by activity staining.

**Results:**

LDH was significantly elevated in cases as well as between the case (mild and severe) groups, showed a moderate positive statistically significant correlation with systolic, diastolic blood pressure and a sensitivity of 50% and a specificity of 80%. Further, the isoenzyme pattern showed a decreasing distribution of aerobic forms of LDH in preeclampsia-eclampsia.

**Conclusions:**

Serum total LDH may serve as a robust and affordable marker of preeclampsia. Serum total LDH, along with its isoenzyme profile, might serve as a predictor and a stronger marker of preeclampsia when compared to serum LDH analysis alone. It may also be used to assess the severity of preeclampsia and hence help in predicting and preventing adverse maternal and foetal outcomes.

## Abstract (sr)

**Uvod:**

Preeklampsija, multisistemski sindrom koji brzo napreduje zbog trudnoće, vodeći je uzrok morbiditeta i mortaliteta majki i novorođenčadi. Ova studija ima za cilj da proceni nivo ukupne laktat dehidrogenaze u serumu kod žena sa preeklampsijom u poređenju sa normotenzivnim trudnicama i proceni elektroforetski obrazac LDH izoenzima u normalnoj trudnoći, preeklampsiji i eklampsiji.

**Metode:**

Studija sprovedena na Odeljenju za biohemiju Medicinskog fakulteta MVJ obuhvatila je 30 pacijentkinja sa preeklampsijom i 30 normotenzivnih trudnica u istom stadijumu trudnoće primljenih na odeljenje OBG. Ukupni LDH u serumu je analiziran DGKC metodom. Uzorci seruma i pupčane vrpce za analizu raspodele izoenzima prikupljeni su od pacijentkinje sa normalnom trudnoćom koja je bila na porođaju, žene s blagom eklampsijom, dve žene s eklampsijom, i analizirani su vertikalnom elektroforezom na tankom sloju gela, a detekcija je vršena merenjem odgovarajućih reakcija.

**Rezultati:**

LDH je bio značajno povišen u svim grupama, kao i kad se rezultati grupa međusobno porede (blaga i teška preeklampsija), i pokazao je umereno pozitivnu i statistički značajnu povezanost sa sistolnim i dijastolnim krvnim pritiskom, osetljivost od 50% i specifičnost od 80%. Dalje, obrazac izoenzima pokazao je smanjenu distribuciju aerobnih oblika LDH u preeklampsiji-eklampsiji.

**Zaključak:**

Ukupni LDH u serumu može poslužiti kao čvrst i pristupačan marker preeklampsije. Ukupni LDH u serumu zajedno sa njegovim izoenzimskim profilom može poslužiti kao prediktor i bolji marker preeklampsije u poređenju sa analizom LDH u serumu. Takođe se može koristiti za procenu ozbiljnosti preeklampsije i tako pomoći u predviđanju i sprečavanju štetnih ishoda po majku i plod.

## Introduction

Preeclampsia is defined as a pregnancy-specific multi-systemic syndrome of widespread endothelial malfunction and vasospasm developing after 20 weeks of gestation [1] [2] [3]. It is a rapidly progressive condition traditionally defined by increased blood pressure (140/90 mm Hg), fluid retention and proteinuria [4]. The ACOG revised guidelines define preeclampsia as a de-novo and abrupt onset persistent hypertension associated with proteinuria or pathological edema or thrombocytopenia or impaired liver or kidney function or new onset of cerebral or visual disturbances [1] [5]. Eclampsia is defined as preeclampsia with sudden development of seizures or coma during the gestational or postpartum period, non-attributable to other neurological diseases that justify the convulsive state [6]. Globally, preeclampsia is a leading cause of maternal and infant illness and mortality claiming up to 76,000 maternal and 500,000 infant deaths per year, according to conservative estimates [7]. In India, the incidence of preeclampsia is 8% to 10 % among pregnant women [8].

The major challenge posed by preeclampsia is its sudden and acute onset often without definitive symptoms. The major symptoms of preeclampsia, such as rise in blood pressure (BP), severe headache, nausea, vomiting, blurring of vision, light sensitivity, are highly nonspecific. Multifactorial risk factors like nulliparity, multifetal gestations, obesity, diabetes mellitus, maternal age above 35 years are associated with preeclampsia [9]. Hence risk assessment remains an enigma, and a delay in diagnosis often leads to severe maternal and neonatal complications encompassing IUD, foetal growth restriction, preterm birth, placental abruption, HELLP syndrome, eclampsia, maternal coma and even death.

Preeclampsia is characterised by disturbed trophoblastic migration of maternal spiral arteries leading to increased uteroplacental vascular resistance and dysfunction, resulting in reduced intervillous blood flow, oxygen and nutrient deprivation to the foetus [10] [11]. The central mechanism of preeclampsia revolves around placental under perfusion, associated hypoxia and cellular death, emphasizing the pivotal role of lactate dehydrogenase (LDH: an intracellular cytoplasmic enzyme of glucose metabolism, a key indicator of anaerobiosis and cell death) in the pathophysiology of preeclampsia.

Hence, this study aims to assess the serum total LDH levels in preeclampsia and to investigate the differential maternal serum and cord blood LDH isoenzyme profile in preeclampsia-eclampsia.

## Materials and Methods

This cross-sectional study was carried out in MVJ Medical College and Research Hospital. The study was approved by the Institutional Ethical Committee, and informed consent was obtained from all study participants.

### Patients

Sixty women admitted to the Department of Obstetrics and Gynaecology in MVJ Medical College and Research Hospital were recruited for the study. Thirty women with preeclampsia constituted the case group. 30 normotensive gestational age-matched (20 weeks or more) pregnant women admitted to the antenatal ward were recruited as controls. Preeclampsia was defined as per the standard guidelines (Blood pressure >/=140/90 mm Hg on at least two occasions, six hours apart and/or proteinuria (>/=300mg/24 hours or >/=1+ dipstick) after 20 weeks of gestation). Known cases of chronic hypertension, any renal disease, hypothyroidism, hyperthyroidism, any metabolic disorder prior to pregnancy were excluded from the study. Participants with preeclampsia were further divided into mild and severe preeclampsia, based on systolic and diastolic blood pressure (Mild preeclampsia: SBP 140 mm Hg -159 mm Hg or BP 90 mm Hg -109 mm Hg with/ without proteinuria of ≤ 300 mg/day, severe preeclampsia: SBP 160 mm Hg or diastolic BP 110 mm Hg, with severe proteinuria and/or signs and symptoms of target organ damage [2]).

An apparently healthy full-term pregnant woman, a woman with mild preeclampsia and two women with diagnosed eclampsia were recruited as participants for the evaluation of LDH isoenzyme profile.

### Biochemical analysis

Serum total LDH was analysed by Deutsche Gesellschaft für Klinische Chemie (DGKC) method. Samples for LDH electrophoresis were preserved in neatly labelled aliquots, stored at -80 °C, coded to ensure blindfolding and sent for further analysis of isoenzyme profile to BioEra Life Science Pvt Ltd where the samples were analysed by 7.5% native polyacrylamide slab gel electrophoresis at a pH of 8.8 followed by LDH activity staining. The isoenzyme profile of lactate dehydrogenase protocol by Department of Medical Biochemistry, Semmelweis University was used as a reference method. The pattern so obtained was analysed with the help of GelAnalyzer (Version 2010a, by Istvan Lazar, http://www.gelanalyzer.com/) software to study the isoenzyme distribution in each of the samples.

### Statistical analysis

Group differences were evaluated using independent sample t-test for quantitative characteristics. P-value less than 0.05 is considered statistically significant. Spearman's rank correlation coefficient test was used to ascertain with relationships between various parameters. The data were analysed using GraphPad Prism (Version 5.00, March 2007) and MedCalC (https://www.medcalc.org).

## Results

The study included 60 women in their third trimester of pregnancy (Control: n = 30; Preeclampsia: n = 30). The preeclampsia group was further classified into mild (n = 16) and severe (n = 14) preeclampsia based on their blood pressure recorded at the time of admission. Table 1 provides the details of maternal age, gestational age and blood pressure in the three groups.

**Table 1:** Group characteristic differences between Control, Mild Preeclampsia and Severe Preeclampsia

	Control Group (n = 30)	Mild Preeclampsia Group (n = 16)	Severe Preeclampsia Group (n = 14)
Maternal Age (Years)	23.63 ± 2.65	24.19 ± 4.56	24.64 ± 4.53
Gestational Age (weeks)	34.7 ± 5.62	36.38 ± 4.05	35.14 ± 4.64
Systolic BP (mm Hg)	113.07 ± 7.71	144.13 ± 4.92	173.57 ± 13.93
Diastolic BP (mm Hg)	70.87 ± 6.80	93.75 ± 4.67	107.14 ± 8.25

**Figure 1:**
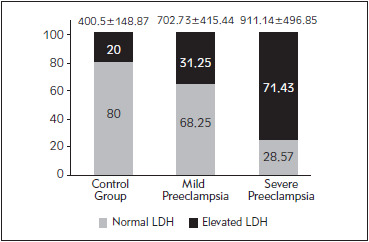
Comparison of total LDH levels and percentage elevations shown in Controls, mild preeclampsia and severe preeclampsia Note: Figure represents the percentage of participants showing elevated total LDH in each group

The control group had a mean serum LDH of 400.5 ± 148.87 U/L while the preeclampsia group showed a mean serum LDH of 702.73 ± 415.44 U/L (p-value = 0.0006). A statistically significant elevation was also observed within the two groups with mild preeclampsia showing serum LDH levels of 520.38 ± 209.09 U/L, while the severe preeclampsia group had LDH values of 911.14 ± 496.85 U/L (p-value = 0.007). Figure 1 represents the percentage of participants showing elevated serum LDH in the control, mild preeclampsia and severe preeclampsia groups.

Serum LDH showed a moderate positive correlation with both SBP (r = 0.549, p < 0.0001) and DBP (r = 0.546, p < 0.0001). A ROC for LDH showed an AUC of 0.793 (95% CI: 0.668 -0.888, p-value < 0.0001), sensitivity of 50%, specificity of 80%, a positive predictive value of 70% and a negative predictive value of 61.54% (Cut off: 525 IU/l, calculated from https://www.medcalc.org).

**Figure 2:**
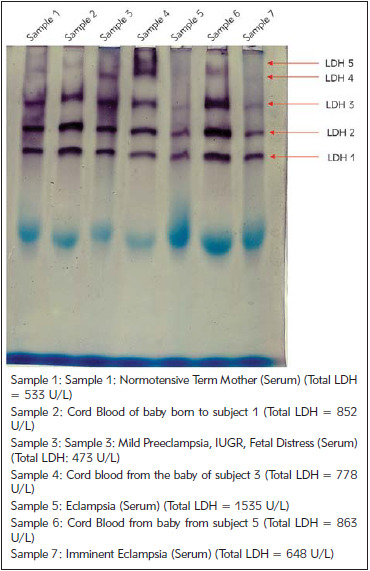
Zymogram showing LDH isoenzyme distribution

Further, the isoenzyme pattern distribution of the maternal serum and cord blood was assessed. Figure 2 shows the LDH isoenzyme distribution of the samples (Total LDH levels indicated in the box below figure).

Bands representing the isoforms, LDH 1, LDH 2 and LDH 3 are clearly seen in all the samples while LDH 4 and LDH 5 isoforms are either lightly stained or undetected in most samples. Sample 1 and sample 4 show all the five isoenzymes, sample 1 shows faint bands of LDH 4 and LDH 5 bands while all the bands are sharp and clear in sample 4.

**Figure 3:**
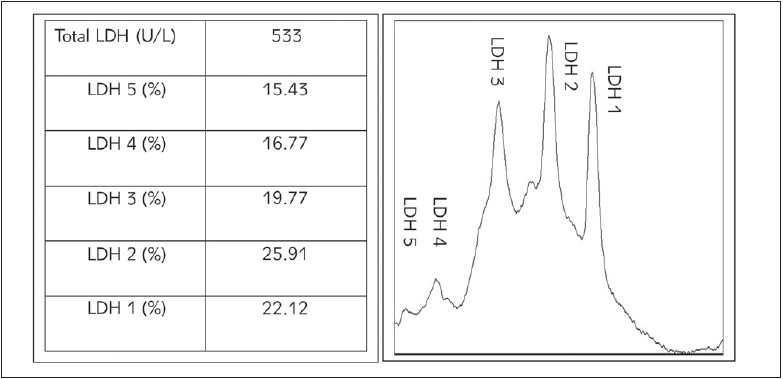
Total LDH and isoenzyme pattern of a normotensive healthy term woman (Sample 1) Note: Table at the left represents the percentage contribution of each LDH isoenzyme in the normal control maternal serum. (Percentage contribution of each isoenzyme is calculated based on GelAnalyzer generated raw volumes.) Graph (right) represent the graphical density recording of the percentage contribution of each isoform to the total LDH.

Figure 3 represents the total LDH and the percentage contribution by each isoenzyme in the maternal serum of the control.

Table 2 shows the fold changes of the isoenzyme in the cases when compared to the control normal maternal serum and cord blood. Pattern changes of the isoforms in the maternal serum of the cases when compared to the control (Sample 1: Maternal serum of a normal term pregnant women undergoing delivery) and that in the cord blood of the cases when compared to the control (Sample 2: Cord blood of the baby born to the normal term pregnant women) are presented.

**Table 2:** Fold Change of LDH isoenzymes in the cases when compared to control Note: Left end represents the Fold Changes of the isoforms in the maternal serum of the cases when compared to the control (Sample 1: Maternal serum of a normal term pregnant women undergoing delivery). Right end represents the Fold Changes of the isoforms in the cord blood of the cases when compared to the control (Sample 2: Cord blood of the baby born to the normal term pregnant women). Fold changes calculated based on GelAnalyzer generated raw volumes.

Maternal serum				Cord Blood	
Isoform distribution changes in maternal serum of cases compared to control maternal serum				Isoform distribution changes in cord blood of cases compared to control cord blood	
Isoforms	Sample 3	Sample 5	Sample 7	Sample 4	Sample 6
LDH 5	–	–	–	–	–
LDH 4	1.02	–	–	1.08	1.26
LDH 3	0.90	0.79	0.86	0.57	0.70
LDH 2	0.72	0.80	0.70	0.66	0.68
LDH 1	0.84	0.83	0.85	0.88	0.73

The results of the isoenzyme distribution clearly indicate a significant decrease in LDH 1, LDH 2 and LDH 3 in preeclampsia and eclampsia. LDH 4 distribution in the cord blood, is seen to be increased in preeclampsia -eclampsia compared to a normotensive normal pregnancy. LDH 5 did not show a clear band in most of the samples, and hence, its pattern alteration could not be studied with our analysis. The trends in maternal serum LDH 1, LDH 2 and LDH 3 levels appear consistent with the pattern changes observed in the respective cord blood sample.

## Discussion

The most plausible explanations of the pathogenesis of preeclampsia focus on the placenta [7]. The initial event of placentation involves the formation of the non-invasive trophoblastic shell, which progresses into an invasive phenotype, causing an exponential rise in the entry of oxygenated maternal blood into the intervillous space [11]. The opening of intervillous space and the consequent increase in PO2, reduces HIF-1 alpha expression, augmenting tropho-blastic differentiation along the invasive pathway [11]. Preeclampsia progresses through a proposed twostage model that includes poorly perfused placentation (Stage 1) arresting trophoblastic invasion, increasing vascular resistance and subsequent nutrient deprivation which modifies maternal metabolism to increase nutrient availability; inability to tolerate this modification leads to the clinical manifestations of preeclampsia (Stage 2) [12]. Lactate Dehydro-genase (LDH), a predominantly intracellular cytoplasmic enzyme of anaerobic glycolysis, is released to the general circulation during cell death and may be increased in preeclampsia due to vigorous glycolysis and chronic anoxemia as a result of placental ischemia. Further, LDH A gene is a well-characterised hypoxia-inducible gene and elevation/ineffective downregulation of HIF-1 alpha upregulates LDH A expression [13]. Hence, LDH may be a prime candidate marker of ischemia, tissue damage associated with the endothelial dysfunction and the severity of preeclampsia.

In the present study, the control group showed serum LDH well within the recommended cut off of 525 U/L, while women with preeclampsia showed statistically significant elevation. The difference in serum LDH between mild and severe preeclamptic groups was also found to be statistically significant. Majority of the normotensive pregnant women had serum LDH within the normal range while a large percentage of preeclamptic women showed elevated levels with serum LDH showing a sensitivity of 50% and a specificity of 80%.

These findings are in agreement with many previous studies. Studies show high serum LDH levels correlate well with the severity of the disease and outcomes in patients of preeclampsia [10] [14] [15]. A study carried out by Mary et al. [16] also suggests increased serum levels of LDH, uric acid and liver enzymes in severe preeclampsia and infers that LDH values greater than 800 U/L correlates with increased risk of perinatal mortality. Further, a study by Purnima and Sonal and another study by Munde et al. [18] also concludes that LDH can be effectively used as a biochemical marker as it reflects the severity of preeclampsia and may be helpful in its effective management [17] [18].

A predominantly intracellular cytoplasmic enzyme of anaerobic glycolysis, LDH, catalyses the interconversion of pyruvate to lactate and is stimulated by hypoxia [19]. It is composed of ‘H’ and ‘M’ chains coded by two different genes (LDH – A coding M chain & LDH – B coding H chain) that combine in various combinations to form five isoenzymes: LDH 1 (H4), LDH 2 (H3M1), LDH 3 (H2M2), LDH 4 (H1M3) & LDH 5 (M4) with their unique tissue-specific distribution depending on the tissue oxygen availability [20] [21]. A small amount of tissue breakdown causes significant elevation of serum LDH since LDH activities are 500 – 700 times higher in most tissue than in general circulation [20]. Heterogeneity of LDH activity seen in electrophoretically separated homogenates/samples from various tissues indicate a characteristic distribution of isoenzyme activity in each site/tissue [22]. Thus, total LDH is only a nonspecific marker of cellular damage while determining the selective distribution of isoenzyme pattern may reflect functional differences in LDH isoenzyme activity related to energy metabolism and oxygen availability and helps in the differential diagnosis of pathologic states [22].

The isoenzyme profile of the maternal serum from the normotensive term control (sample 1) showed predominant contributions of LDH 1 (22.1%) and LDH 2 (25.9%) forms with LDH 3, LDH 4 and LDH 5 contributing 19.8%, 16.8% and 15.4% respectively. A study by Neal et al. [20] on serum LDH profile and uterine preparedness for labour, reports an almost similar serum isoenzyme distribution pattern with LDH 1 contributing 29.66%, LDH 2 of 30.33%, LDH 3 of 19.21%, LDH 4 of 8.74% and LDH 5 of 12.07% at labour. Our analysis of isoenzyme profiles suggests decreased levels of aerobic forms of LDH in preeclampsia when compared to normotensive pregnancy and also hints that the distribution changes in maternal serum are concordant with the changes in the cord blood (the closest representative of placental biology and metabolism) isoenzyme distribution. This could indicate that the isoenzyme perturbations might give a better picture of impending preeclampsia -eclampsia, even when the total serum LDH values do not show significant elevations and remain inconclusive. The hypothesis that hypoxia induces the LDH A gene which leads to increased production of the more anaerobic forms of LDH in the preeclampsia and eclampsia reported by the previous study thus supports our observation of a decrease in aerobic forms of LDH.

A study by Tsoi et al. [21] 21 concludes that expression of LDH A gene is increased in the endothelial cells of the placenta and hence increased LDH 5 isoenzyme activity is a marker of endothelial pathology in preeclampsia. The study further explains that LDH -A gene is a well-characterised hypoxiainducible gene and the upregulation of its expression combined with the shift of LDH profile to a more anaerobic side supports the proposed role of hypoxia in preeclampsia placenta and upregulation of HIF-1 alpha [21]. Makkonen et al. [13] indicated that LDH 2 was decreased while LDH 3 was elevated in severe preeclampsia. In Sammour et al. [23], LDH 5 was significantly increased in both serum and placental extracts in severe preeclampsia when compared to normal pregnancy.

To conclude, the study clearly shows the association of elevated LDH with preeclampsia. LDH is shown to significantly correlate with the severity of the disease. LDH shows a positive correlation with systolic and diastolic BP and a moderately fair specificity and sensitivity. The isoenzymes profile indicates that preeclampsia and eclampsia are associated with a shift in the pattern and decrease in the aerobic forms of the enzymes, probably due to induction of LDH A gene and/or repression of LDH B gene by hypoxia, pattern changes in maternal serum appear concordant with changes in cord blood, indicating the maternal isoenzyme pattern to be a specific and reliable predictor of the developing hypoxia even when total LDH values seem inconclusive. Further, total LDH along with the isoenzyme distribution of LDH may serve as a more robust marker and/or predictor of the hypertensive disorders of pregnancy. Assessment of LDH, when combined with routine markers might thus aid in early risk assessment, close monitoring and prompt management of the disease.

A definitive conclusion of these findings needs further validation. A cross-sectional study design and a small sample size can be seen as the limitations of our study. The study needs to be replicated in a larger prospective model to obtain a clearer picture of the association of these parameters with the development and severity of preeclampsia.

*Acknowledgements*. We are immensely grateful to the Principal and Management of MVJMC & RH, to the HOD, Department of Biochemistry and all the staffs of Biochemistry (teaching &technical) and OBG Departments (nursing staff) of the institution for providing us with the platform, encouraging us and supporting us throughout the study. Our special thanks to Late Dr S R Gurumurthy, for supporting us through all our ventures and being a constant source of encouragement. We also express our immense gratitude to the team of BioEra Life Science Pvt Ltd, Pune, Maharashtra, with special thanks to Dr Vishwadeep Kapare, Technical Expert and Ms Sneha Pradhan for their relentless support and cooperation during our brief collaboration. Finally, we express our sincere gratitude to the ICMR STS team for encouraging us by approving the study for ICMR STS 2018 (Reference ID: 2018-02850).

## Conflict of interest statement

The authors state that they have no conflicts of interest regarding the publication of this article.

## List of abbreviations

LDH, Lactate Dehydrogenase; ACOG, The American College of Obstetricians and Gynecologists; SBP, Systolic blood pressure; DBP, Diastolic blood pressure; IUGR, Intrauterine growth retardation; HIF, Hypoxia Inducible Factor; TNF, Tumour Necrosis Factor.
